# Functionality and Neurocognition in Patients With Bipolar Disorder After a Physical-Exercise Program (FINEXT-BD Study): Protocol of a Randomized Interventionist Program

**DOI:** 10.3389/fpsyt.2020.568455

**Published:** 2020-10-29

**Authors:** Saínza García, Ilargi Gorostegi-Anduaga, Edurne García-Corres, Sara Maldonado-Martín, Karina S. MacDowell, Cristina Bermúdez-Ampudia, María J. Apodaca, Irene Pérez-Landaluce, Ignacio Tobalina-Larrea, Juan C. Leza, A. González-Pinto

**Affiliations:** ^1^Severe Mental Illness Research Group, Bioaraba Health Research Institute, Vitoria-Gasteiz, Spain; ^2^Department of Psychiatry, Osakdietza Basque Health Service, Araba University Hospital, Vitoria-Gasteiz, Spain; ^3^Centre for Biomedical Research Network on Mental Health (CIBERSAM), Madrid, Spain; ^4^School of Medicine, University of the Basque Country (UPV/EHU), Vitoria-Gasteiz, Spain; ^5^Department of Physical Education and Sport, Faculty of Education and Sport, University of the Basque Country (UPV/EHU), Vitoria-Gasteiz, Spain; ^6^GIzartea, Kirola eta Ariketa Fisikoa Ikerkuntza Taldea (GIKAFIT), Society, Sports, and Physical Exercise Research Group, University of the Basque Country (UPV/EHU), Vitoria-Gasteiz, Spain; ^7^Department of Pharmacology and Toxicology, Faculty of Medicine, Complutense University of Madrid, Madrid, Spain; ^8^Epidemiology and Public Health Research Group, Bioaraba Health Research Institute, Vitoria-Gasteiz, Spain; ^9^Department of Cardiology, Osakdietza Basque Health Service, Araba University Hospital, Vitoria-Gasteiz, Spain; ^10^Department of Ophthalmology, Osakdietza Basque Health Service, Araba University Hospital, Vitoria-Gasteiz, Spain; ^11^Department of Nuclear Medicine, Osakdietza Basque Health Service, Araba University Hospital, Vitoria-Gasteiz, Spain

**Keywords:** bipolar disorder, physical exercise, intervention program, neurocognition, functional capacity

## Abstract

**Introduction:** Recent studies have shown that symptoms of psychiatric illness, functionality, and cognitive function improve with exercise. The aim of this study will be to investigate whether the implementation of an individualized exercise program will improve the functional status of patients with bipolar disorder (BD).

**Methods:** This longitudinal, interventional, randomized, controlled, simple-blind clinical trial will include 80 patients aged 18–65 years, all of them with BD diagnosis. Patients will be randomly assigned to a physical exercise intervention + Treatment-As-Usual (TAU) group and a non-intervention + TAU group. Patients will be assessed by an extensive battery of clinical tests, physical parameters (e.g., brain structure changes measured by optical coherence tomography, cardiorespiratory fitness) and biological parameters (inflammation, oxidative stress and neurotrophic factors) at baseline, after a 4-month intervention period, and 6-month follow-up.

**Discussion:** This is an innovative study aimed at gaining a deeper understanding of the physiopathology of BD and determining whether the prognosis and evolution of the disease can be improved through modifiable areas of the patient's lifestyle.

**Clinical Trial Registration:** NCT04400630. NCT clinicaltrials.gov. Date of registration in primary registry 22 May 2020. clinicaltrials.gov.

## Introduction

Bipolar disorder (BD) is a mood disorder affecting about 400 million people worldwide (1). This psychiatric disease is a chronic disorder associated with cognitive dysfunction, increased mortality and intensive use of healthcare services (2, 3). The management of BD involves a combination of pharmacological therapies and psychosocial interventions including, but not limited to, cognitive-behavioral therapy, functional remediation, family psychoeducation, social skills training and protected employment (4). Despite therapies, the risk of recurrence is high, and patients often do not recover completely. Failure of current therapies to induce the remission of BD symptoms has led researchers to focus their efforts on developing more effective treatments (5).

In the search for new therapeutic strategies, physical exercise has been found to play a relevant role in the treatment of psychiatric disorders (6). Thus, physical exercise is not only effective in improving cardiorespiratory function, physical status, and metabolic syndrome (common comorbidities in BD), but it has also proven to reduce depressive symptoms and suicidal ideation (7).

Several studies have documented that moderate-to-vigorous physical exercise intensity programs influence the progression of mood disorders (8) and also improves cognitive performance and functionality in patients with schizophrenia (9). Concerning these results, at a biological level, physical exercise increases levels of neurotransmitters, cortisol, beta-endorphins, and neurotrophic factors such as BDNF (brain-derived neurotrophic factor) (8). It has been shown that serum levels of BDNF are reduced in persons with BD (10, 11) and major depressive disorder (12, 13). This neurotrophic factor plays a key role in neurological development, adult brain plasticity, neuron survival and differentiation, neuronal function, repair mechanisms of brain plasticity, and stimulates neurogenesis after the activation of its TrkB receptor (9). This receptor has two isoforms: a functional isoform with TrKB-FL activity, and a *truncated* isoform without TrkB-T activity (14). Elevated TrkB-T levels may induce neuronal death by the inhibition of the functional TrkB-FL isoform. In this line, patients with schizophrenia have been reported to exhibit increased levels of the *truncated* isoform in the prefrontal cortex (15). Indeed, TrkB-T levels in peripheral blood are more strongly associated with the early stages of psychosis than BDNF levels (14).

Physical exercise also modulates the body's response to stress and optimizes its antioxidant and anti-inflammatory capacity. Since BD patients have been reported to have an inflammatory state at the peripheral and central nervous system (16), the anti-inflammatory effects of physical exercise could play a relevant role in the physiopathology of the disease (17).

Interaction between the mechanisms described above explains how physical exercise can reduce symptoms of mood disorders and recurrence rates, thereby improving the quality of life and functionality of patients (18).

In line with the goal of better understanding the physiopathology of psychiatric diseases, brain imaging has been used for 15 years to assess brain alterations associated with these disorders. In BD patients, gray matter shrinks in the hippocampus, the fusiform gyrus, and the temporal lobe. The severity of brain alterations are related to symptoms and the number of episodes experienced by the patient (19). Patients with schizophrenia also show a loss of brain volume even since the first psychotic episode and in prodromal stages (20). Several authors have postulated that the retina could be an accessible marker of the structural and/or functional integrity of the brain (21–23). This hypothesis is grounded on the fact that the retina develops from the same embryonic layer as the brain. The retina and the brain are connected via the optic nerve, which would allow direct observation of the brain (22). Retinal alterations might be concomitant to the inflammatory and neurodegenerative processes and structural central nervous system alterations associated with BD (24). The retina is considered to be a part of the brain and contains several layers of neurons that are connected by synopses. The retinal nerve fiber layer (RNFL) is composed of unmyelinated axons of nodal cells. The ganglion cell complex contains the three innermost layers of the retina and comprises the retinal nerve fiber layer, the ganglion cell-inner layer, and the inner plexiform layer. Optical coherence tomography (OCT) is a non-invasive, no-contact, rapid imaging technique without known side effects that provides high-resolution and cross-sectional images of the retina, and measures RNFL (25). Some studies have reported OCT abnormalities such as retinal layer thinning (also in RNFL) associated with different neurological disorders with degenerative changes, including Parkinson's disease (26), Alzheimer's disease (27), and multiple sclerosis (28). Connecting with the growing interest in this technique for the diagnosis of neurodegenerative diseases, a recent study revealed that OCT demonstrated a thinning of retinal layers in patients with schizophrenia and schizoaffective disorder, as compared to a group of healthy individuals (29).

Previous studies have shown that physical exercise is an effective antidepressant intervention (30), but no studies have been able to provide a comprehensive overview of the mechanisms that produce these benefits in terms of patient prognosis. This fact led us to conduct a longitudinal comparative study to assess the impact on FunctIonality and Neurocognition of an individualized moderate-to-vigorous physical EXercise Training in a population of patients with BD (FINEXT-BD study).

We hypothesize that this intervention will improve the functional status of patients and this improvement will be reflected in physical and biological parameters. Thus, the primary aim of this study will be to investigate whether the implementation of an individualized moderate-to-vigorous physical exercise intensity program as adjuvant therapy to standard drug therapy will improve the functional status of patients with BD, as compared to a group of patients with BD without intervention. The specific secondary objectives of the study will be to: (1) investigate whether the implementation of this exercise program modifies the cognition, the clinical symptoms and incidence of comorbidities; (2) investigate whether the implementation of this exercise program induces changes in the levels of oxidative and inflammatory parameters and these changes correlate with the evolution of clinical symptoms; (3) whether BDNF and TRkB-FL/TRkB-T ratio are associated with the evolution of the patient's functional and neurocognitive capacity, and (4) whether changes in the thickness of retinal layers as measured by OCT correlate with the functionality and neurocognitive function of BD patients.

## Methods and Analysis

### Design

The FINEXT-BD study is a longitudinal, interventional, randomized, controlled, simple-blind trial conducted at the University Hospital of Araba in a cohort of BD patients divided into two groups: (1) physical exercise intervention + Treatment As Usual (TAU) (intervention group) and (2) patients with no intervention + TAU (TAU group). In addition, a gender-, race-, and age-matched healthy control group will be added to compare biological and physical parameters and the functional and neuropsychological status, with the cohort of BD patients.

Treatment As Usual will be defined as the prescribed pharmaceutical treatment by the clinician at the dose indicated for euthymic patients, with regular monthly visits that can be increased to weekly or biweekly sessions in the presence of prodromal symptoms or relapse.

#### Randomization and Blinding

FINEXT-BD patients will be randomly assigned to the groups (1:1) using Random Allocation Software version 1.0.0 software. Study evaluators will be blinded to the treatment branch. Patients will continue their usual treatment and will be treated by their reference psychiatrist, who will be informed of patient's participation in the interventionist program and will make sure that the appropriate clinical treatment is administered.

**Figure 1:**
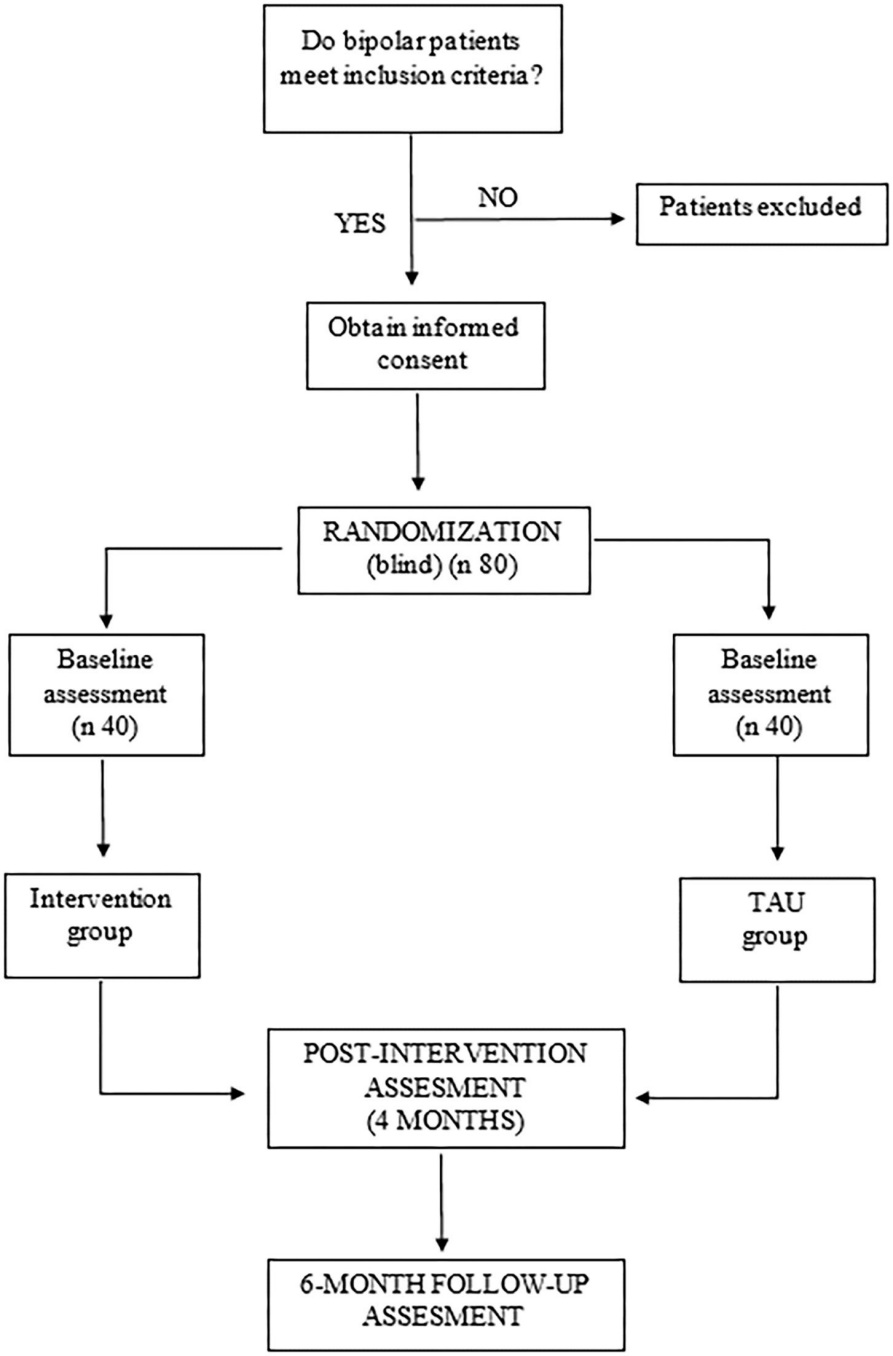
FINEXT-BD study procedure.

The flow diagram of the study can be seen in Figure 1.

### Participants

#### Patients

The sample of patients with BD will be recruited by trained and experienced psychiatrists from patients treated in the Inpatient and Outpatient Unit of Psychiatry of the University Hospital of Alava.

Inclusion criteria will be:

1. Diagnosis of BD according to the Diagnostic and Statistical Manual of Mental Disorders, Fifth Edition (DSM-5) (31), diagnosis based on semi-structured SCID-P interview (32). The study will involve patients with first-episode or multiple episodes of mania. Patients will be required to be euthymic at baseline, defined as no current diagnosis of an episode of mania, hypomania, or depression. Relapse during follow-up or presence of subsyndromal symptoms at any moment will not be exclusion criteria.
2. Age between 18 and 65 years.
3. Speak Spanish correctly.

Exclusion criteria will be:

1. Intellectual disability (assessed by DSM-V).
2. History of cranial trauma with loss of consciousness.
3. Physical diseases that cause mental health problems.
4. Pervasive developmental disorders.
5. Pregnancy or breastfeeding.
6. Be classified as “physically active” according to the Global Physical Activity Questionnaire (GPAQ) (33).
7. The presence of a severe or uncontrolled cardiovascular risk factor such as unstable coronary artery disease, uncontrolled hypertension, malignant ventricular arrhythmia, atrial fibrillation, exercise-induced ischemia, and ventricular failure during exercise.
8. Other significant medical conditions including, but not limited to chronic or recurrent respiratory, gastrointestinal, neuromuscular, or any musculoskeletal problems interfering with exercise.
9. The presence of inflammatory diseases.
10. Consumption of anti-inflammatory drugs during the week prior to blood extraction.

#### Controls

Controls will be recruited from friends of patients and healthcare personnel. If the established N is not reached, informative posters will be placed in different hospitals. In such case, posters will be sent to the CEIC for evaluation.

The inclusion criteria will be:

1. Age between 18 and 65 years.
2. Speak Spanish correctly.

The exclusion criteria will be:

1. Intellectual disability (assessed by DSM-V).
2. History of cranial trauma with loss of consciousness.
3. Physical diseases that causes mental health problems.
4. Pervasive developmental disorders.
5. Pregnancy or breastfeeding.
6. Consumption of anti-inflammatory drugs during the week prior to blood extraction
7. The presence of inflammatory diseases.
8. History of psychiatric episodes among first-degree relatives.

### Assessments

Data collection will be based on an assessment protocol for gathering data on sociodemographic, physical, clinical, and biological variables. Patients from the two groups will be evaluated by blind raters at baseline (pre-intervention), after a 4-month intervention period, and 6-month follow-up period. Blood sampling for the analysis of biological parameters will be performed during the same visits. The control group will be evaluated only at baseline.

#### Sociodemographic Variables

Data on sociodemographic variables (age, sex, educational level, socioeconomic level, employment status, family history of psychiatric disorders, number of relapses, years of disease evolution, and number of manic episodes) will be collected at baseline.

#### Physical Parameters

The following physical parameters will be assessed at each of the three visits at the Psychiatric Service of the Alava University Hospital (HUA): body mass (kg) with individuals in light clothing and barefoot using a portable scan body composition and scale (Omron KARADA); height (cm) with participants in a standing position with no shoes using a measuring tape with shoulders in a relaxed position; body mass index will be calculated by dividing body mass to height (kg/m^2^); waist circumference (cm) will be measured using a flexible anthropometric tape midway between the iliac crest and lower rib margin; blood pressure (mm Hg) will be measured in the right arm with the patient in a seated and relaxed position by using a life-support monitor (General Electric carascape U100 Dinamap technology); and a blood sample will be drawn after 12 h overnight fasting to quantify serum levels of triglycerides, glucose and high-density lipoprotein cholesterol. With all this information, the diagnosis of metabolic syndrome will be documented in accordance with the National Cholesterol Education Program Adult Treatment Panel III (ATP III) (34). Thus, patients will have metabolic syndrome if they meet at least two of the following criteria: (1) waist circumference ≥ 102 cm in men or ≥ 88 cm in women; (2) serum triglycerides ≥ 150 mg/dl (8, 3 mmol/L); (3) cholesterol HDL <40 mg/dl (2, 2 mmol/L) in men and <50 mg/dl (2, 8 mmol/L) in women; (4) systolic blood pressure ≥130 mmHg or diastolic blood pressure ≥85 mmHg; (5) serum glucose ≥100 mg/dl (5, 6 mmol/L).

The Ophthalmology Service of the HUA will carry out the study of retinal layers by OCT (HRA+OCT Spectralis, version 6.0, Heidelberg Engineering, Germany): RNFL, GCL and IPL thickness in the two eyes, with each layer divided into segments: overall, temporal, nasal, superior and inferior. In the HUA cardiology service, patients will perform the cardiopulmonary exercise test (CPET) at the baseline visit and after the intervention. At these same visits, in the Nuclear Medicine Service of the HUA, a Dual-energy X-ray absorptiometry (Hologic QDR 4500 W) will be used to evaluate the body composition of participants. This whole-boy scan will be performed to analyze the amount of body fat and muscle mass of participants.

#### Physical Fitness

Physical fitness includes a peak, symptom-limited CPET. The CPET will be performed on a treadmill T-2100 (General Electrics Healthcare, Germany) in the cardiology department of the Alava University Hospital. The testing protocol will start with 1.6 km/h with a 0% slope, with gradual increments of 0.1 km/h and a 1% slope every 15 s to exhaustion with continuous electrocardiogram monitoring. During the test, participants will be verbally encouraged by the nurse in charge of the test. The expired gas analysis will be conducted using a commercially available system (Ganshorn, Germany) that will be calibrated before each test with a standard gas of known concentration and volume. Breath-by-breath gas exchange data will be measured continuously during exercise and averaged every 60 s. Peak oxygen uptake is defined as the highest oxygen uptake (VO_2peak_) value attained toward the end of the test. Achievement of true peak effort can be assumed in the presence of two or more of the following criteria: (1) volitional fatigue (>18 on BORG scale), (2) peak respiratory exchange ratio (VCO2/VO2) ≥1.1, (3) achieving >85% of age-predicted maximum heart rate (HR), and (4) failure of VO_2_ and/or HR to increase with further increases in work rate (35). A self-reported Borg rating of perceived exertion (6–20 scale) will be recorded at the end of the test. Blood pressure will be measured every 2 min throughout the test. Ventilatory thresholds (i.e., VT1 and VT2) will be assessed by standardized methods using the V-slope and ventilatory equivalents. First ventilatory threshold (VT1) will be identified as the point of transition in the carbon dioxide production (VCO_2_) vs. VO_2_ slope from <1 to >1, or VT1 is identified as the nadir of the ventilatory equivalent (VE) of VO_2_ vs. work rate relationship. The second ventilatory threshold (VT2) is identifiable as the nadir of the VE/VCO_2_ vs. work rate relationship (35). Absolute and relative indications for terminating the exercise test will be taken into account (36). The identification of the two VT will determine the three different exercise intensity domains or ranges for exercise design (R1, R2, R3): (R1) light to moderate exercise intensity with HR values below VT1; (R2) moderate to high or vigorous exercise intensity with HR values between VT1 and VT2, and (R3) high to severe intensity exercise intensity with HR values up to VT2 to peak intensity. When it is not possible to identify the VT2, exercise intensity domains are established taking into account percentages of HR reserve, i.e., moderate intensity is defined between 50 and 75% of HR reserve, high intensity from ≥76% to <95% of HR reserve (35).

#### Clinical Variables

##### Diagnosis

Patients will be diagnosed according to the DSM-5 criteria using the semi-structured SCID-P interview (32).

Analysis of the pre-morbid adjustment of patients as part of a determination of their prognosis will be made using an *abbreviated version of the Phillips Scale of Pre-morbid Adjustment* (37). This scale is an abbreviated version of the original five-section Phillips Pre-morbid Rating Scale and is composed of two parts: Part I (Abbreviated Pre-morbid Sexual Rating Scale) and Part II (Abbreviated Pre-morbid Personal-Social Rating Scale). This scale uses a 7-point scale (0–6) to evaluate each part.

##### Psychosocial functioning

The functioning of patients will be measured using *the Functioning Assessment Short Test (FAST*) (38). This scale is a brief instrument designed to assess functional impairment in psychiatric disorders. The scale comprises 24 items that cover six specific areas of functioning: autonomy, occupational functioning, cognitive functioning, financial issues, interpersonal relationships, and leisure time. Each item is scored in a 0–3 points range (0: no difficulty; 1: mild difficulty; 2: moderate difficulty; 3: severe difficulty) with total score ranging from 0 to 72 points (39).

The patient functionality will be assessed through the *Global Assessment of* Functioning (GAF) (40). This scale rates the level of functioning and severity of symptoms on a 0–100 scale, with higher values corresponding to better overall functionality.

Strauss-Carpenter scale (41). This scale will be employed to assess the psychosocial functioning of patients and consists of four items rated from 0 to 4 on a Likert-type scale and yields a total score that is calculated by the addition of all item scores: the higher the score is, the better the prognosis (42).

##### Neurocognition

- *The Stroop Color and Word Test (SCWT)* (43): is a neuropsychological test that will be used to assess selective attention and the impulsivity domain, executive subject's ability to inhibit cognitive interference that occurs when the processing of a specific stimulus feature impedes the simultaneous processing of a second stimulus attribute, well-known as the Stroop Effect. Individuals are required to read three different tables as fast as possible. Two of them represent the neutral condition in which participants are required to read aloud a list of color name words printed in black ink and name different color patches. In contrast, in the third table, the conflict condition is provided by having color words written in an incongruous color of ink and participants are required to name the color of the ink instead of reading the word (44).
- The Complutense Verbal Learning Test (TAVEC) (45): The TAVEC is the Spanish version of the California Verbal Learning Test (CVLT) (46) and will be used for the assessment of different memory and learning processes, such as immediate recall, short and long term memory with and without semantic clues and recognition (47).
- The Computerized version of Wisconsin Card Sorting Test (WCST) (48): neuropsychological test that assesses individuals' executive function. WCST will be used to measure the subject's capacity to deduce concepts and apply a strategy to adapt behavior to changing conditions (49). This test consists of cards depicting simple geometric figures that vary in color, shape, and number. Examinees must sort cards in accordance with one of three viable rules: color, shape, or number of the depicted object(s) (50).

##### Clinical severity

- *The Clinical Global Impression Scale (CGI)* (51) will be used to assess symptom severity, global improvement and therapeutic response. This scale consists of two subscales that evaluate, respectively the clinical severity and the overall improvement in the patient's symptomatology. Each subscale has a single item scored between 0, not evaluated, and 7 which corresponds to the maximum severity (severity subscale) or to a state of no improvement and aggravated worsening of the symptomatology (improvement subscale).
- The presence of suicidal thoughts or behaviors will be assessed by means of *the Likert Clinical Global Impressions–Severity of Suicide scale (CGI-SS)* (52). This scale scores from 1 to 5 the patient's current risk of suicide.

##### Illness awareness

The illness awareness of patients will be measured using *the Scale to assess Unawareness in Mental Disorders (SUMD)* (53). This scale explores the thoughts and beliefs of patients regarding their illness and its pharmacological treatment. SUMD scale comprises nine items (current awareness of the following states): (1) a mental disorder; (2) consequences of a mental disorder; (3) effects of drugs; (4) hallucinatory experiences; (5) delusional ideas; (6) disorganized thoughts; (7) blunted affective; (8) anhedonia, and (9) lack of sociability. Each item is scored as follows: not applicable (response of “0” or missing data), aware (response of “1”), slightly aware/unaware (response of “2”), and seriously unaware (response of “3”) (54).

##### Medication adherence

- Adherence to medication will be assessed using the *Morisky-Green scale*. This self-reported scale includes four yes/no questions rated on a 0–4 scale, considering patients who obtain 0 points as adherent and those who obtain a 1+ score as non-adherent (55).
- Response to lithium will be measured by the *Retrospective Criteria of Long-Term Treatment Response in Research Subjects with Bipolar Disorder (Alda scale)* (56). This scale was specifically developed to evaluate the long-term mood stabilization effect under naturalistic conditions. Briefly, this scale quantifies the degree of improvement during the treatment (Criterion A), which is rated on a 0–10 scale, and weighs clinical factors that are considered to be relevant for determining if the observed improvement is a result of the treatment rather than a spontaneous improvement or an effect of additional medication (Criteria B1-B5), which are rated as 0, 1, or 2 points. The total score on the Alda scale is obtained by subtracting the B score from the A score (57).

##### Clinical symptomatology

- Depressive symptoms will be measured by *the Hamilton Rating Scale for Depression (HRSD*) (58). This scale consists of 17 items, each of which offers between 3 and 5 possible answers with values ranging from 0–2 or 0–4 points, respectively. The total score ranges from 0 to 52 points, and different cut-off points are used to classify the patient's depressive symptoms. Those who obtain scores of 0–7 are identified as non-depressed, minor depression 8–12, moderate depression 13–17, severe depression 18–29, and those who obtain scores above 30 are characterized as very severe depression (59).
- Manic symptoms will be measured using *the Young Mania Rating Scale (YMRS)* (60). This scale is composed of 11 items, each item consisting of 5 answer choices that are scored on a 0–4 scale, except four of the 11 items that are scored with double points (0, 2, 4, or 8 points). The total score ranges from 0 to 60, with the status of euthymia being classified as getting 6 or fewer points, hypomania as getting 12 or more points, and mania as getting between 20 and 60 points.

##### Quality of lifestyle

- *The World Health Organization Quality of Life-BREF (WHOQOL-BREF) instruments* will be used to measure the quality of life. It assesses the individual's perceptions in the context of their culture and value systems, as well as their personal goals, standards, and concerns. The WHOQOL-BREF instruments comprise 26 items that measure the following broad domains: physical health, psychological health, social relationships, and environment (61).
- The severity of substance addiction will be measured with the *European version of the Addiction Severity Index (EuropASI)*, a semi-structured interview, which measures the severity of addiction in different areas: medical, employment, alcohol consumption, use of other drugs, legal problems, family and social relationships, and psychological state (62).
- Chronobiological rhythms will be assessed with the *Biological Rhythms Interview of Assessment in Neuropsychiatry (BRIAN)* (63). This scale contains 21 items designed to assess five domains related to biological rhythms: (1) Sleep, (2) Activities, (3) Social aspects, (4) Alimentation, based on the last 15 days, and (5) predominant Rhythm (chronotype) based on the last year. The total score may range from 16 to 84, with the higher the score obtained, the greater the alteration of the biological rhythms (64).

#### Biological Variables

Venous blood samples (10 mL) will be collected between 8:00 and 9:00 after overnight fasting by the nursing staff in polypropylene EDTA-containing tubes. Fresh blood will be stored at 4°C until processing about 1 h. later. Blood will be centrifuged (652 g × 10 min, 4°C); the resulting plasma samples will be collected and stored at −80°C until use. The rest of the sample will be diluted 1:1 in culture medium (RPMI 1640, GIBCO) and a gradient with Ficoll-Paque (GE Healthcare) will be used to isolate peripheral blood mononuclear cells (PBMC) by centrifugation [800 g × 40 min, room temperature (RT)]. The PBMC layer will be aspired and suspended in RPMI and centrifuged at 1800 g for 15 min, RT. The supernatant will be removed and the PBMCs pellet will be stored at −80°C until analysis.

##### Biochemical measurements in plasma

- *Nitrite (*${NO}_{2}^{-}$*):* The final and stable product of nitric oxide, will be measured in plasma using the Griess method (65). Briefly, in an acidic solution with 1% sulphanilamide and 0.1% N-(2-napthyl) ethylenediamine dihydrochloride (NEDA), nitrites will be converted into a pink compound that will be measured photometrically at 540 nm in a microplate reader (Synergy 2, BioTek).
- *Lipid Peroxidation (TBARS):* In the process of lipid peroxidation, there are several intermediate and final products due to the degradation of cell membranes, among which is malondialdehyde (MDA), used as a marker of cellular damage. The Thiobarbituric Acid Reactive Substances (TBARS) test is the most used for the measurement of this metabolite in plasma where thiobarbituric acid (TBA) reacts with malondialdehyde (MDA) under high temperature (95°C) and acidic conditions to produce a MDA-TBA complex that is measured photometrically at 530 nm (Synergy 2, BioTek) (66). The results obtained through this procedure will be expressed in μM of MDA.
- *Total Antioxidant Status (TAS):* This parameter reflects the cumulative effect of all antioxidants present in plasma and is determined by a standardized spectrophotometric assay. This assay is based on the ability of the antioxidants present in the sample to inhibit the oxidation of ABTS (2,2'-azino-bis-[3-ethylbenzothiazolin-6-sulfonic]) to the ABTS+ cation radical by the action of methemyoglobin (peroxidase). The concentration of this radical is measurable by spectrophotometry at 600 nm, the result of which is inversely proportional to the total antioxidant level of the sample (67).
- *Prostaglandin Levels*: PGE2 plasma levels will be measured by a competitive colorimetric immunoassay in which the PGE2 present in the sample competes with the PGE2-linked conjugate (provided by the kit) for binding to the anti-PGE2 antibody present in the wells of the plate. Then the substrate is added which triggers a measurable enzymatic reaction at 412 nm (Synergy 2, BioTek). Since the concentration of the conjugated is constant, the intensity of the bound yellow color is inversely proportional to the concentration of PGE2 in the sample.
- *Cytokine levels:* Plasma levels of IL-6 will be measured by enzyme immunoassays EIA kits, a technique based on recognition by specific antigen-antibody binding, both cytokines will be measured by spectrophotometry at 450nm in a microplate reader (Synergy 2, BioTek).
- *Glutathione peroxidase (GPx)* catalyzes the reduction of hydroperoxides, including H_2_O_2_, by reduced glutathione and functions to protect the cell from oxidative damage. The measure of GPx activity is based on a coupled reaction with glutathione reductase (GR). Oxidized glutathione (GSSG) produced upon reduction of hydroperoxide by GPx is recycled to its reduced state by GR and NADPH. The oxidation of NADPH to NADP+ is accompanied by a decrease in absorbance at 340 nm. Under conditions in which the GPx activity is rate-limiting, the rate of decrease in the absorbance is directly proportional to the GPx activity in the sample.
- *Glutathione (GSH)* serves as a nucleophilic co-substrate to glutathione transferases in the detoxification of xenobiotics and is an essential electron donor to GPx in the reduction of hydroperoxides. The oxidized GSH dimer (GSSG) is formed from GSH and H_2_O_2_ by the GPx. The detection of both forms, GSH and GSSG, could be detected using comercial kits, a colorimetric substance reacts with the free thiol group on GSH to yield a highly colored product that can be measured at 405 nm (Synergy 2, BioTek). On the other hand, by using 2-vinylpyridine to block any free GSH in the sample GSSG can be determined, because any sample that has not been treated with 2-vinylpyridine will yield Total GSH levels. Then, the free GSH concentration can be calculated from the difference between the total GSH and GSSG.
- *BDNF levels:* An *in vitro* enzyme-linked immunosorbent assay will be used for the quantitative measurement of human BDNF in plasma. This assay employs an antibody specific for human BDNF coated on a 96-well plate. Standards and samples are pipetted into the wells and BDNF present in a sample is bound to the wells by the immobilized antibody. A secondary antibody HRP-conjugated is used for the detection of bound BDNF. Then, a TMB substrate solution is added to the wells and color develops in proportion to the amount of BDNF bound, the absorbance is measured at 450 nm (Synergy 2, BioTek).

##### Biochemical measurements in PBMCs

To carry out all biochemical determinations, PBMC samples will be first fractionated in cytosolic and nuclear extracts. For the preparation of cytosolic and nuclear extracts, it will be used a modified procedure based on the Schreiber et al. (68). PBMC pellets will be homogenized in 150 μL of lysis buffer consisting of 10 mmol/L HEPES at pH 7.9, with a cocktail of proteases and phosphatase inhibitors (Roche), 1 mmol/L EDTA, 5 mmol/L NaF, 1 mmol/L NaVO4, 0.5 mol/L sucrose and 10 mmol/L Na2MoO4. After 15 min the detergent Nonidet P-40 (Roche, Mannheim, Germany) will be added at a concentration of 1%. The tubes will be shaken for 30 s and the mixture will be centrifuged for 5 min at 8,000 g. The supernatant that constitutes the “cytosolic fraction” will be aspirated and the pellet will be resuspended in 50 μL of buffer supplemented with 20% glycerol and 0.4 M KCL. The mixture will be stirred for 30 min at 4°C, centrifuged for 5 min at 13.000 g and the supernatant, corresponding to the nuclear fraction, will be aspirated. Both fractions were stored at −80°C.

###### Protein Quantification

Once the nuclear extracts have been obtained, protein quantification will be carried out. The Bradford^©^ assay is one of the most popular methods to determine the concentration of a total protein in a sample. In this assay, proteins bind to Coomassie G-250 dye resulting in a color change from brown to bright blue and the formation of a protein-dye complex. The absorbance of the blue color produced is measured at 570–590 nm and is proportional to the protein concentration. Known bovine serum albumin concentrations with seven points between 0 and 0.8 mg/ml were used as the standard curve. For the measurements, the samples were diluted 1/20 and mixed with 10 μl of dilution or the standard with 200 μl of Bradford reagent and the absorbance was measured in the plate reader (Sinergy 2, BioTek, Germany).

*TrkB-FL and TrkB-T receptors:* Protein levels of receptors were quantified by Western blot analysis. In brief, 12.5 μg of cytosolic extracts were loaded onto electrophoresis gels. Protein samples were separated and transferred onto nitrocellulose membrane (Transfer Pack, Biorad) using a semi-dry transfer system and were blocked in 5% BSA for 1.5 h, and then the membranes were incubated overnight at 4°C with specific antibodies. Blots were imaged using an Odyssey® Fc System (Li-COR Biosciences) and quantified by densitometry (ImageJ®, NIH). We used β-actin as a loading control. All densitometry results are expressed as a percentage of the control. Given the counterbalancing effect of TrkB-T1 and TrkB-FL, we chose the ratio of TrkB-FL to TrkB-T expression (hereafter FL/T ratio) as our index variable for describing BDNF receptor expression.

### Exercise Intervention Program

Previous investigations by the exercise specialists who will lead this research have already performed the exercise protocol (69, 70). In short, the experimental group will receive supervised physical exercise treatment by high-intensity interval training (HIIT), alternating high and moderate intensities (20 min). Participants will exercise two non-consecutive days per week for 16 weeks under the supervision of a coach at the facilities provided by the University Hospital of Araba. All the exercises sessions will start and finish with blood pressure monitoring and training intensity will be controlled by HR monitoring (Polar M200, Kempele, Finland) and through the rate of perceived exertion using the Borg's original scale (6–20 point). Each session will include a 10 min warm-up with joint mobility and coordination exercises with continuous leg movement to facilitate the venous return and a 10 min cool-down period with basic core strengthening exercises and passive stretching exercises on the floor to ensure a progressive return to the resting values of both blood pressure and HR. The main portion of the training session will consist of 20 min of aerobic exercises on the bike developing progressively intensity. Intensity will be individually tailored to HR at moderate or vigorous intensities, adjusting the power and speed on the bike, to achieve the planned target HR (Table 1). The exercise specialists will keep detailed records of all the exercise sessions reporting the HR and Borg scale values of every interval. The importance of targeting moderate and high intensity will be emphasized.

**Table 1:** Intervention program for the experimental group by High-intensity physical exercise program on the bike.

	**Protocol**			
	**High-intensity interval**		**Moderate intensity interval**	
**Weeks**	**Volume (min)**	**Intensity (% heart rate reserve)**	**Volume (min)**	**Intensity (% heart rate reserve)**
1–2	2	80	18	60
3–4	3	80	17	60
5–6	4	85	16	65
7–8	4:30	85	15:30	65
9–10	4:30	95	15:30	70
11–12	4:30	95	15:30	70
13–16	4:30	95	15:30	70

*Volume and intensity progression*.

Several strategies will be implemented to maximize adherence, including music in all sessions, individualized attention at the intervention sessions and telephone calls following missed sessions.

The protocol of HIT will carry out a 5–10-min warm-up period on the bike. After that participants will cycle for 30 s at high-intensity (i.e., HR values up to VT2 to peak intensity) followed by 60 s at moderate-intensity (i.e., HR values between VT1 and VT2). Four repetitions (1 rep = 30 s high-intensity followed by 60 s moderate-intensity) will be initially performed and gradually increased to nine repetitions will be completed. The training session will end with a 5–10 min cool-down period at moderate-intensity.

### Statistical Analysis

A general descriptive analysis of the sample will be performed to assess baseline homogeneity. The normality of quantitative variables will be assessed by the Kolmogorov-Smirnov test and results will be expressed as means and standard deviation or as median values and interquartile range in the case of non-normal distribution. Qualitative variables will be expressed as frequencies and percentages.

To respond to the main aim, analysis of covariance (ANCOVA) will be used to assess the improvement in functionality. The same test will be used to analyze the difference between the groups.

At baseline, an analysis of variance (ANOVA) will be performed to confirm that there is no difference between patient groups, but there is a difference with the control group.

Student's *t*-test for related samples will be used to assess changes after the physical exercise program between intervention patient groups, among normally distributed variables. Changes in the variables studied across the three visits will be assessed by an analysis of repeated measurements. The analysis of repeated measurements will be used to assess the evolution of the variables studied during the three visits. Non-normally distributed variables will be assessed using Wilcoxon test. Univariate and multivariate regression analysis will be performed to determine whether statistically significant differences are associated with the evolution of the functionality and clinical and cognitive symptoms. All statistical analyses were carried out using SPSS v23.0 and R v3.5.0 statistical software, with the significance level set at *p* < 0.05.

#### Sample Size

To calculate the sample size, a review of the literature related to the main topic of the study was carried out and using the Ene 2.0. computer program, it was determined that to obtain a power of 90% and an effect size of 0.65 with a significance of 5% and with possible losses to follow-up of 10% of the patients, it is necessary to have a sample of at least 38 patients per group to detect differences between the means of the main variables (EEAG and FAST). Therefore, we have estimated a sample size of 40 per group.

## Anticipant Results

This study anticipates that the patients belonging to the intervention group, at the end of the individualized physical exercise program, will show an improvement of their functional capacity, as compared to the TAU group. In addition a decrease in the clinical symptoms of the disease, as well as a better score on the neuropsychological scales is expected. Furthermore, completion of the intervention program will have greatly increased adherence to treatment in these patients. If we confirm our hypothesis, all these improvements will be reflected at the biological substrate level (decrease in oxidative and inflammatory parameters and increase in BDNF) and physical level (changes in retinal structure and anthropometrics) in the post-intervention and follow-up visits, compared to the control group.

Further, this program of physical exercise performed regularly for 16 weeks will provide patients with guidelines to improve their lifestyle and quality of life, which will be reflected in the post-intervention and follow-up visit at 6 months after the end of the intervention program.

## Dissemination

The final database obtained will be the property of the research team and shall not be shared without the principal investigator's permission. This protocol and the results obtained after the completion of the study will be presented at national and international conferences as well as published in scientific journals.

## Discussion

One of the most important objectives that Psychiatry is trying to address new strategies that improve clinical outcomes in patients. In this sense, the randomized single-blind intervention study described in this article aims to investigate whether the implementation of an individualized physical exercise program as an adjuvant therapy will improve the prognosis of patients with BD, by improving their functionality.

Previous studies have shown that physical exercise in people without psychiatric disease had protective effects against depression (71). In patients with major depression, it has been observed that physical exercise reduces symptoms of depression (72). In addition, in another study in these patients, spatial working memory improved with high-dose exercise, while other cognitive domains including attention, visual memory, and spatial planning improved regardless of the dose of exercise (73). Another research including a subset of patients with BD and schizophrenia found that intense circuit training improved memory, processing speed, and symptoms of depression (74). Therefore, there is broad variability in the response of individuals with psychiatric diseases to physical exercise and how it influences the prognosis of each patient (75). As functionality is related with cognition, and cognitive deficits are related with subthreshold depressive symptoms in BD patients (76), it is possible to hypothesize that positive effects of physical activity on functionality are related also with mood stabilization in patients with psychiatric disorders such as BD. That is why it is so important to study the effect of physical activity on functionality of patients diagnosed with BD and the real mechanisms by which exercise influences functionality, considering both clinical symptoms as subthreshold depressive, manic symptoms, and cognitive symptoms, and physiopathology. Other therapies have shown that non-pharmacological treatments improve functionality and cognition (77), and that BDNF is related with this improvement (78).

To our knowledge, no holistic studies have been performed so far to investigate how moderate-to-vigorous physical exercise influences cognitive deterioration and functional capacity in patients with BD using clinical scales, neuroinflammatory and oxidative stress parameters, and by the study of changes in brain structure.

Our study is subject to a limitation typical of longitudinal studies: loss of follow-up either by voluntary discontinuation of the participant or by a relapse of the patient during the study. Nevertheless, the number of patients receiving treatment or with first episode BD who are lost to follow-up will be limited, as their treatment will involve regular follow-up visits at University Alava Hospital. Another aspect to consider is the representativity of the sample. The results obtained will only correspond to BD patients 18–65 years of age and in a sample of 40 participants per group. But, an advantage of the University Hospital of Alava is that it is the only emergency care center of the region, and also the only center that attends patients with episodes of BD. The severity of illness in patients with long-standing disease is heterogeneous, and they may be treated in Outpatient or Partial Inpatient units.

## Conclusion

This is an innovative study aimed at gaining a deeper understanding of the physiopathology of BD and investigating how the prognosis and evolution of the disease can be improved through potential modifiable areas of patients' lifestyle. Moreover, with the results of this study it will be possible to provide patients with solid tools for managing their disease, making them co-managers of their own illness, which would also increase their awareness of the disease and ultimately their adherence to treatment.

## Ethics Statement

This study was approved by the local Ethics Committee, the University Hospital of Alava Ethics Committee (September 20, 2019, Certificate No. 2019-036) in accordance with the Declaration of Helskinki II. After providing written and oral information about the study, informed consent was obtained from all participants. This study is registered with the international standard randomized controlled trial NCT04400630.

## Author Contributions

SG and AG-P wrote the first draft of the manuscript. AG-P, SG, and EG-C participated in the clinical design of the intervention. SM-M and IG-A participated in the design of the exercise intensities and the physical-exercise program. KM, JL, and SG participated in the design of the biochemical measurements. CB-A participated in the design of the statistical analysis. All authors participated in the drafting of the manuscript and all of them approved the final version.

## Conflict of Interest

AG-P had received grants and served as consultant, advisor or CME speaker for the following entities: Janssen-Cilag, Lundbeck, Otsuka, Pfizer, Sanofi-Aventis, Alter, Angelini, Exeltis, the Spanish Ministry of Science and Innovation (CIBERSAM), the Ministry of Science (Carlos III Institute), the Basque Government, the Stanley Medical Research Institute. The remaining authors declare that the research was conducted in the absence of any commercial or financial relationships that could be construed as a potential conflict of interest.
